# Gal4-based Enhancer-Trapping in the Malaria Mosquito *Anopheles stephensi*

**DOI:** 10.1534/g3.112.003582

**Published:** 2012-11-01

**Authors:** David A. O’Brochta, Kristina L. Pilitt, Robert A. Harrell, Channa Aluvihare, Robert T. Alford

**Affiliations:** * Institute for Bioscience and Biotechnology Research, University of Maryland, College Park, Rockville, Maryland 20850; † Department of Entomology, University of Maryland, College Park, Rockville, Maryland 20850; ‡ Insect Transformation Facility, University of Maryland, College Park, Rockville, Maryland 20850

**Keywords:** malaria, Plasmodium, Aedes, dengue, Drosophila

## Abstract

Transposon-based forward and reverse genetic technologies will contribute greatly to ongoing efforts to study mosquito functional genomics. A *piggyBac* transposon-based enhancer-trap system was developed that functions efficiently in the human malaria vector, *Anopheles stephensi*. The system consists of six transgenic lines of *Anopheles stephensi*, each with a single *piggyBac-Gal4* element in a unique genomic location; six lines with a single *piggyBac-UAStdTomato* element; and two lines, each with a single *Minos* element containing the *piggyBac*-transposase gene under the regulatory control of the *hsp70* promoter from *Drosophila melanogaster*. Enhancer detection depended upon the efficient remobilization of *piggyBac-Gal4* transposons, which contain the yeast transcription factor gene *Gal4* under the regulatory control of a basal promoter. *Gal4* expression was detected through the expression of the fluorescent protein gene *tdTomato* under the regulatory control of a promoter with *Gal4*-binding *UAS* elements. From five genetic screens for larval- and adult-specific enhancers, 314 progeny were recovered from 24,250 total progeny (1.3%) with unique patterns of *tdTomato* expression arising from the influence of an enhancer. The frequency of *piggyBac* remobilization and enhancer detection was 2.5- to 3-fold higher in female germ lines compared with male germ lines. A small collection of enhancer-trap lines are described in which *Gal4* expression occurred in adult female salivary glands, midgut, and fat body, either singly or in combination. These three tissues play critical roles during the infection of *Anopheles stephensi* by malaria-causing Plasmodium parasites. This system and the lines generated using it will be valuable resources to ongoing mosquito functional genomics efforts.

Vector-borne diseases, such as mosquito-transmitted malaria, dengue fever, and filariasis, among many others, not only remain health threats to a significant fraction of the world’s population but also significantly impact the economies of countries in which there is intense transmission (World Health Organization 2010). In the case of malaria, controlling the mosquito vectors of malaria-causing *Plasmodium* parasites continues to be a major component of malaria control efforts. Understanding the genetic and molecular genetic basis of insecticide resistance, olfaction, reproductive physiology and the immune system of *Anopheles* mosquitoes figures heavily into contemporary ideas for developing new strategies for controlling mosquito populations and *Plasmodium* transmission (Carey *et al.* 2010; Catteruccia 2007; Enayati and Hemingway 2011; Alonso *et al.* 2011).

Recent advances in mosquito molecular genetics have depended upon the availability of whole-genome sequence data and a host of technological advances, including transcription-profiling and RNA-based gene-silencing technologies (Blandin *et al.* 2002; Dimopoulos *et al.* 2000; Holt *et al.* 2002). However, powerful functional genomics technologies for finding and mutating mosquito genes as well as regulating transgene expression, such as enhancer- and gene-trap technologies, have been lacking.

Transposons can be used as platforms upon which some of these powerful functional genomics technologies can be constructed. Transposon-based enhancer detection is an effective way to sense the presence of enhancers and when coupled to robust binary transcription regulatory systems such as the Gal4 system, the “trapped” enhancers can be used to regulate the expression of any transgene under the regulatory control of a promoter containing *Gal4* upstream activation sequences (*UAS*) without having to physically isolate and characterize the regulatory elements (Brand and Perrimon 1993). Gene traps enable genes to be detected based on the patterns of expression of transgenes carried on the transposon, and in many cases, transposon integration results in disabling the target gene (Stanford *et al.* 2001). The resulting recessive hypomorphic or null mutations can be of great value in efforts to determine a gene’s function. The power of these technologies and the myriad variations that exist are particularly well displayed in many studies of the popular animal model systems *Drosophila melanogaster* and *Mus musculus* (Bellen 1999; Duffy 2002; Friedel and Soriano 2010) and to a lesser extent in “nonmodel” systems (Awazu *et al.* 2004, 2007; Balciunas *et al.* 2004; Kontarakis *et al.* 2011; Lorenzen *et al.* 2007; Trauner *et al.* 2009; Uchino *et al.* 2008). Vector biologists could benefit substantially from the availability of these technologies for the study of mosquitoes.

Transposon-based transgenic technologies have been available for mosquitoes for over a decade but they are utilized somewhat infrequently because the creation of primary transgenic mosquitoes can be technically challenging and because some transposons, once integrated, have shown little or no remobilization activity, severely limiting their utility as functional genomics tools. In *Aedes aegypti*, the transposons *Hermes*, *Mos1*, and *piggyBac*, although effective as vectors for creating transgenic mosquitoes, cannot be remobilized or are remobilized rarely in the presence of functional transposase following their integration into the genome of this species (O’Brochta *et al.* 2004; Sethuraman *et al.* 2007; Wilson *et al.* 2003). Similar observations were made in *Anopheles stephensi* concerning the *Minos* transposon (Scali *et al.* 2007). Consequently, vector biologists have been unable to develop powerful transposon-based gene-finding and analysis technologies. Fortunately, the remobilization behavior of *piggyBac* elements integrated into the genome of *Anopheles stephensi* is quite different from that of *Minos*; *piggyBac* is highly active in *An. stephensi* in the presence of transposase, permitting the development of a variety of much-needed gene-finding and analysis technologies in this species (O’Brochta *et al.* 2011).

Here we report on the creation and performance of a *Gal4*-based enhancer-trap system for *An. stephensi*. We show that enhancers are readily detected with our system and that this technology can be used to create lines of mosquitoes with patterns of *Gal4* expression particularly useful for regulating the expression of transgenes in cells and tissues relevant to the study of mosquito/parasite interactions.

## Material and Methods

### Mosquitoes

*Anopheles stephensi* were grown at 29° (80% relative humidity for adults), and larvae were provided with pulverized fish food (TetraMin Tropical Flakes) *ad libitum*, while adults were provided with 10% sucrose continuously. Adult females were occasionally allowed to feed on adult mice to obtain a blood meal, which was necessary for reproduction. The use of mice was with the approval and oversight of the Institutional Animal Care and Use Committee (IACUC) of the University of Maryland, College Park, operating under the National Institutes of Health’s Office of Laboratory Animal Welfare guidelines. Mosquito blood-feeding protocols involving mice were not terminal, and animal pain and distress were minimized with the use of anesthetics with the approval of the IACUC.

#### SDA 500:

This is a wild-type strain of *An. stephensi* originally isolated in Pakistan and selected in the laboratory for susceptibility to *Plasmodium falciparum* infection (Feldmann *et al.* 1990).

#### UMITF-PB-F2^DsRed^ and UMITF-PB-M5^DsRed^:

**Figure 1:**
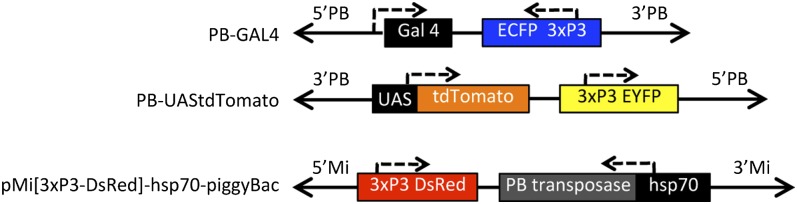
Organization of *piggyBac* and *Minos* vectors. *PB-GAL4* has the *Gal4* ORF (“Gal4”) located just 3′ of the promoter for the *piggyBac* transposase. *piggyBac* sequences containing the 5′ and 3′ inverted terminal repeats and sub-terminal sequences are shown (black arrows; “5′PB” and “3′PB”). This element contains the *ECFP* gene under the regulatory control of a central nervous tissue-specific promoter (“3xP3ECFP”). *PB-UAStdTomato* contains the inverted repeats and sub-terminal sequences of *piggyBac* (black arrows; “5′PB” and “3′PB”), the *EYFP* gene under the regulatory control of a central nervous tissue-specific promoter (“3xP3EYFP”) and the ORF of *tdTomato* under the regulatory control of a minimal promoter with five optimized GAL4 binding sites (“UAStdTomato”) (Brand and Perrimon 1993). pMi[3xP3-DsRed]-hsp70-*piggyBac* is based on the description in Horn *et al.* (2003) and contains the 5′ and 3′ inverted terminal repeats and sub-terminal sequences of *Minos* (black arrows; “5′Mi” and “3′Mi”). This element contains the *DsRed* gene under the regulatory control of a central nervous tissue-specific promoter (“3xP3DsRed”), and the *piggyBac* transposase ORF under the regulatory control of the *hsp70* promoter from *D. melanogaster* (“hsp70PBtransposase”). Dotted lines with arrows show the direction of transcription associated with all transgenes.

These are transgenic lines of SDA 500, with each line containing a single copy of the *Minos* gene vector from p*Mi*[*3xP3-DsRed*]-*hsp70-piggyBac* (Horn *et al.* 2003; O’Brochta *et al.* 2011) (Figure 1). This vector contains the *piggyBac*-transposase open reading frame (ORF) under the regulatory control of the promoter from the *hsp70* gene from *D. melanogaster* (Horn *et al.* 2003). Heat-shock induction was not necessary for expression of *piggyBac* transposase in the germ line or soma of these mosquitoes (O’Brochta *et al.* 2011). We refer here to lines UMITF-PB-F2^DsRed^ and UMITF-PB-M5^DsRed^ as F2 and M5, respectively.

### Vectors

#### PB-GAL4:

This is a *piggyBac* vector with 329 bp of the 5′ terminal sequences and 690 bp of the 3′ terminal sequences of *piggyBac* containing the *Gal4* ORF under the regulatory control of the *piggyBac* transposase gene’s promoter in addition to a visible marker gene encoding the *enhanced cyan fluorescent protein* (*ECFP*) under the regulatory control of the *3xP3* promoter (Berghammer *et al.* 1999). This vector was constructed using Gateway recombination cloning technology (Invitrogen, Grand Island, NY), in which four recombination modules were simultaneously recombined into a destination plasmid. The first module consisted of the first 329 bp of the 5′ terminal sequences of *piggyBac* (GenBank J04364). The second module consisted of the *Gal4* ORF from pGaTB attached to the 3′ UTR of the *hsp70* gene of *D. melanogaster* (Brand and Perrimon 1993). When the first and second modules were joined during site-specific recombination, the *piggyBac* transposase promoter was juxtaposed to the *Gal4* ORF. The third module consisted of *ECFP* under the regulatory control of the *3xP3* promoter, which was isolated from pXL-pBac-ECFP (Berghammer *et al.* 1999; Li *et al.* 2005). Recombination between modules two and three joined the *Gal4* enhancer detector module and the marker gene such that transcription of each was in opposite directions. The fourth module consisted of last 690 bp of the 3′ terminal sequences of *piggyBac* (Figure 1).

#### PB-UAStdTomato:

This is a *piggyBac* vector with 671 bp of the 5′ terminal sequences and 690 bp of the 3′ terminal sequences of *piggyBac* containing the tandem-dimer form of the *DsRed* variant *Tomato* (*tdTomato*) (Shaner *et al.* 2004) under the regulatory control of a promoter with *Gal4*-binding and upstream activating sequences (*UAS*), along with a marker gene consisting of the *enhanced yellow fluorescent protein* (*EYFP*) under the regulatory control of the *3xP3* promoter. This vector was also constructed using Gateway recombination cloning technology (Life Technologies, Grand Island, NY) involving the simultaneous recombination of four recombination modules into a destination plasmid. The first module contained 690 bp of the 3′ terminal sequences of *piggyBac*. The second module contained 1.5 kb of the *tdTomato* ORF from *ptdTomato* (Clontech, Mountain View, CA) inserted into pUAST-attB at the *Eco*RI/*Not*I sites between the promoter region containing five *UAS* elements and the 3′ UTR of *hsp70* from *D. melanogaster*. The third module contained *EYFP* under the regulatory control of the *3xP3* promoter, and the fourth module contained 671 bp of the 5′ terminal sequences of *piggyBac* (Figure 1).

### Mosquito transformation

Transgenic *An. stephensi* were created in the University of Maryland, College Park, Institute for Bioscience and Biotechnology Research’s Insect Transformation Facility (http://www.ibbr.umd.edu/facilities/itf) by injecting preblastoderm embryos of SDA 500 *An. stephensi* with vector-containing plasmids and plasmids expressing *piggyBac* transposase (phsp-PBac) (Handler and Harrell 1999). Vectors and transposase-expressing plasmids were each at 50 ng/microliter in injection buffer (5mM KCl, 0.1mM NaPO4; pH 6.8). Insects developing from injected embryos and surviving to adulthood were pooled according to sex and mated to noninjected SDA 500 adults of the opposite sex. The progeny were screened as larvae for the expression of *ECFP* or *EYFP*, and transgenic individuals were used to establish lines. The *piggyBac* insertion sites were determined using splinkerette-PCR after lines were established (see below), and the DNA sequence of their integration sites were deposited in GenBank (accession numbers JX242566–JX242578)

### Gal4 remobilization crosses and enhancer detection

Approximately 100 *PB-Gal4*–containing individuals (male or female, depending on the cross) were mated *en masse* with ∼100 *piggyBac* transposase-expressing individuals of the opposite sex (*UMITF-PB-M5^DsRed^* and *UMITF-PB-F2^DsRed^*). Approximately 100 individuals heterozygous for both *PB-Gal4* and *UMITF-PB-F2 ^DsRed^* or *UMITF-PB-M5^DsRed^* were mated to ∼100 *PB-UAStdTomato* individuals *en masse*, and the resulting progeny were screened as third or fourth instar larvae and as adults for *tdTomato* expression. Although *piggyBac* transposase was under the regulatory control of the promoter from the *hsp70* gene from *D. melanogaster*, heterozygous individuals containing both *PB-Gal4* and *piggyBac* transposase were not heat-shocked. Earlier work showed that heat shock was unnecessary for transposase expression and *piggyBac* remobilization using these and similar lines (O’Brochta *et al.* 2011). The number of individuals with novel patterns of *tdTomato* expression was recorded, and selected individuals were used to start lines.

### Splinkerette-PCR

The splinkerette-PCR genotyping method is based on amplification of genomic DNA containing the 5′ or 3′ end of the *piggyBac* element and a variable amount of adjoining genomic DNA (Devon *et al.* 1995; Potter and Luo 2010). This method was used to confirm the integration of *piggyBac* into the genome, to compare genotypes of transgenic individuals, and to sequence the genomic DNA flanking integrated *piggyBac* elements to locate the integration site within the genome. Splinkerette-PCR was performed as described previously using genomic DNA isolated from individual third or fourth instar larvae or adults (O’Brochta *et al.* 2011; Potter and Luo 2010).

### Bioinformatics analysis

DNA sequence data obtained from splinkerette-PCR, representing genomic DNA flanking the *piggyBac* enhancer-trap element, was used to query publicly available *An. gambiae* genome sequence data and an assembled draft genome of *An. stephensi* [created and made available by Dr. Zhijian (Jake) Tu at Virginia Polytechnic Institute and State University, Blacksburg, VA 24061, and now publically available on VectorBase (Lawson *et al.* 2007)]. Insertion sites were located to scaffolds within the current *An. stephensi* genome release, AsteV1. All DNA sequence queries were performed using the algorithm basic local alignment search tool (BLAST) (Altschul *et al.* 1990).

### Microscopy

The patterns of *tdTomato* expression were determined by microscopic observations of larvae, pupae, and adults using an Olympus MVX10 fluorescent dissecting microscope equipped with Chroma filters (Chroma Technology Corporation, Bellows Falls, VT) 49001 ET-CFP (excitation, 436/20; emission, 480/40; dichroic, 455), 49002 ET-GFP (excitation, 470/40; emission, 525/50; dichroic, 495), 49003 ET-EYFP (excitation, 500/20; emission, 535/30; dichroic, 515), 49005 ET-DsRed (excitation, 545/30; emission, 620/60; dichroic, 570) as well as a Zeiss Axiom Imager A1 fluorescent compound microscope with Zeiss filter set 20 (excitation, 546/12; emission, 575–640; dichroic, 560) and filter set 38HE (excitation, 470/40; emission, 525/50; dichroic, 495).

## Results

### Transgenic lines

Six independent *Gal4* enhancer-trap–containing lines were created, each with a single *piggyBac* element. Similarly, six *UAStdTomato*-containing lines were created, each of which contained a single *UAStdTomato* transgene (Table 1). The locations of the inserted elements varied, and all integrations involved canonical cut-and-paste transposition into TTAA target sites, as expected when using *piggyBac* transposons (Fraser 2000). The chromosomal locations of integrated elements in *An. stephensi* were assigned to scaffolds in the most current *An. stephensi* genome-release, AsteV1, available on VectorBase (Lawson *et al.* 2007) (Table 1). None of the *PBGal4*-containing lines, with the exception of UMITF-PBGal4.5, had detectable *Gal4* expression and were therefore sensitive reporters of enhancers encountered during element remobilization. Line UMITF-PBGal4.5 had low levels of *Gal4* expression in the central nervous system, including the brain and ventral ganglia, due to the presence of an enhancer near the primary integration site. This element can still be used for enhancer-trap screens, depending on the target phenotypes that are of interest in the screen. None of the *UAStdTomato*-containing lines had detectable *tdTomato* gene expression in the absence of *Gal4*.

**Table 1:** Enhancer-trap system for *Anopheles stephensi*

Line	Location*^a^*	GenBank*^b^*
*UMITF-PBGal4.1*	04796: 42751-54	JX242568
*UMITF-PBGal4.2*	05657: 162381-84	JX242569
*UMITF-PBGal4.3*	03905: 171549-52	JX242570
*UMITF-PBGal4.4*	03863: 483-86	JX242571
*UMITF-PBGal4.5*	ND	
*UMITF-PBGal4.6*	01707: 601922-25	JX242572
*UMITF-UAS:tdT1*	02731: 149811-14	JX242573
*UMITF-UAS:tdT2*	01636: 9998-01	JX242574
*UMITF-UAS:tdT3*	02729: 107840-43	JX242575
*UMITF-UAS:tdT4*	04375: 250069-72	JX242576
*UMITF-UAS:tdT6*	05523: 17863-66	JX242577
*UMITF-UAS:tdT8*	00733: 188438-41	JX242578
*UMITF-PB-F2^DsRed^*	01724: 355056-57	JX242566
*UMITF-PB-M5^DsRed^*	02306: 81725-26	JX242567

ND, not determined (*i.e.* no splinkerette data were obtained).

a The scaffold number in *An. stephensi* genome release AsteV1 in VectorBase (Lawson *et al.* 2007) is followed by the nucleotide coordinates of the TTAA (*piggyBac*) or TA (*Minos*) target sites within that scaffold.

b GenBank accession numbers.

### Frequency of enhancer detection

We screened a total 24,250 larvae and adult progeny for the presence of remobilized *Gal4* enhancer-trap elements resulting in the expression of *UAStdTomato*. These progeny were obtained from five independent crosses involving the use of both *piggyBac*-transposase–expressing lines M5 and F2 (Table 2). As observed in an earlier study, a 2.5- to 3-fold higher rate of *piggyBac* remobilization (enhancer-trap events) was observed in the germ line of females compared with the germ line of males (Table 2) (O’Brochta *et al.* 2011). Overall, the frequency of enhancer detection was approximately one enhancer-trap event per 51 progeny screened (2%) when remobilization occurred in the germ line of females. When remobilization occurred in the germ line of males, the frequency of enhancer detection was approximately one enhancer-trap event per 130 progeny screened (0.8%). We did not observe any significant difference between the remobilization frequencies observed when the two *piggyBac*-transposase–expressing lines F2 and M5 were used (*z* = −2.4; *P* = 0.022). When enhancer-trap events were detected, they were almost always represented by a single individual among the progeny. Of the 317 progeny with *tdTomato* expression, we estimate that most resulted from independent transposition events.

**Table 2:** Gal4/UAS-based enhancer-trap screens in *Anopheles stephensi*

Cross	F1*^a^* ♂	F1 ♀	Screened*^b^*	tdTomato*^c^*	Percent*^d^*
A	*PBGal4.1 / M5*	*UAS:tdT1*	4700	26	0.553*^e^*
B	*UAS:tdT1*	*PBGal4.1 / M5*	5500	92	1.673*^e^*
C	*PBGal4.1 / F2*	*UAS:tdT1*	5500	52	0.945*^f^*
D	*UAS:tdT1*	*PBGal4.1 / F2*	4450	102	2.292*^f^*
E	*UAS:tdT2*	*PBGal4.2 / M5*	4100	45	1.098
Totals			24250	317	1.307

a M5 and F2 refer to *piggyBac* transposase-expressing lines *UMITF-PB-M5^DsRed^* and *UMITF-PB-F2^DsRed^*, respectively. All other lines designations omit the *UMITF* prefix.

b Only insects with *PBGal4* (*3xP3ECFP)* and *UAS:tdT* (*3xP3EYFP*) were counted. Screens were conducted at fourth instar and adult stages.

c Total number of fourth instar larvae or adults expressing *tdTomato*.

d (Number of *tdTomato*-expressing insects ÷ Total number of larvae screened) × 100.

e Proportion expressing *tdTomato* in Crosses A and B were significantly different. *z* = −5.27; *P* < 0.001.

f Proportion expressing *tdTomato* in Crosses C and D were significantly different. *z* = −5.41; *P* < 0.001.

### Somatic activity

**Figure 2:**
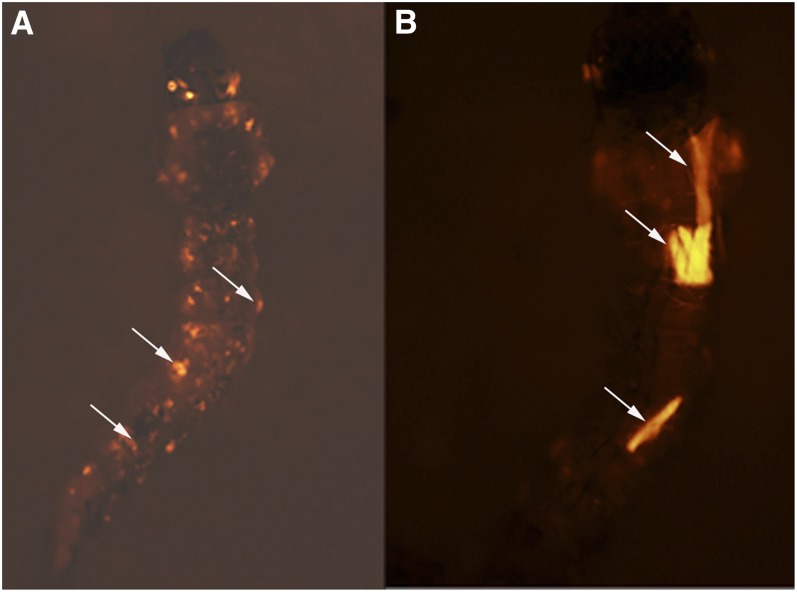
Somatic activity of enhancer-trap elements. (A) Fourth instar larva heterozygous for a *Gal4* enhancer-trap element and the transposase-expressing transgene from the F2 line. The mottled appearance is due to somatic clones of cells (arrows) in which somatic movement of the enhancer-trap element resulted in enhanced expression in subpopulations of larval cells. (B) Fourth instar larva heterozygous for a *Gal4* enhancer-trap element and the transposase-expressing transgene from the M5 line. Enhanced *Gal4* expression was often seen in individual muscles or groups of muscle in asymmetrical patterns that were not heritable, indicating that these were somatic clones. Frequent enhancement of *tdTomato* expression in muscle cells was likely due to the presence of a muscle-specific enhancer near the *piggyBac* transposase-containing transgene in the M5 line, resulting in elevated levels of transposition of the *Gal4* enhancer-trap element in these cells.

The transposase-expressing lines F2 and M5 both expressed *piggyBac* transposase under the regulatory control of the *hsp70* promoter from *D. melanogaster*, and consequently, *piggyBac* remobilization was not expected to be confined to the germ line of insects containing both a *Gal4* enhancer-trap element and *piggyBac* transposase. Indeed, in the F1 heterozygotes containing a *Gal4* enhancer-trap element and a *piggyBac* transposase-expressing transgene, we observed clear evidence of somatic movement of the *Gal4* enhancer-trap element (Figure 2). When the *piggyBac* transposase-expressing transgene originated from the F2 line, the F1 heterozygotes displayed irregular patterns of *tdTomato* expression involving small patches of cells, giving the larvae and adults a distinctly mottled appearance (Figure 2A). These patterns were asymmetrical and not heritable, which is consistent with their somatic nature. When the *piggyBac*-transposase–expressing transgene originated from the M5 line, F1 heterozygotes frequently showed expression of *tdTomato* in individual muscles or groups of muscles in larvae (Figure 2B). The patterns of *tdTomato* expression in the muscles of F1 heterozygotes were also always asymmetrical, and we attribute these patterns to the presence of a muscle-specific enhancer influencing the somatic expression of the *hsp70*-regulated transposase transgene in line M5. We speculate that this results in elevated levels of *piggyBac* transposase in muscle cells, thereby increasing the frequency of remobilization of the *Gal4* enhancer-trap element in these cells and, consequently, the probability of observing *tdTomato* expression.

### Germ line activity

Outcrossing F1 heterozygotes with individuals homozygous for a *UAStdTomato*-containing transgene resulted in the detection of 317 progeny with *tdTomato* expression patterns, consistent with the detection of an enhancer by the *Gal4* enhancer-trap element. Some of these individuals were retained and used to establish permanent lines so they could be used in the future for regulating transgene expression. We describe some of those lines here.

#### UMITF-C2F8:

**Figure 3:**
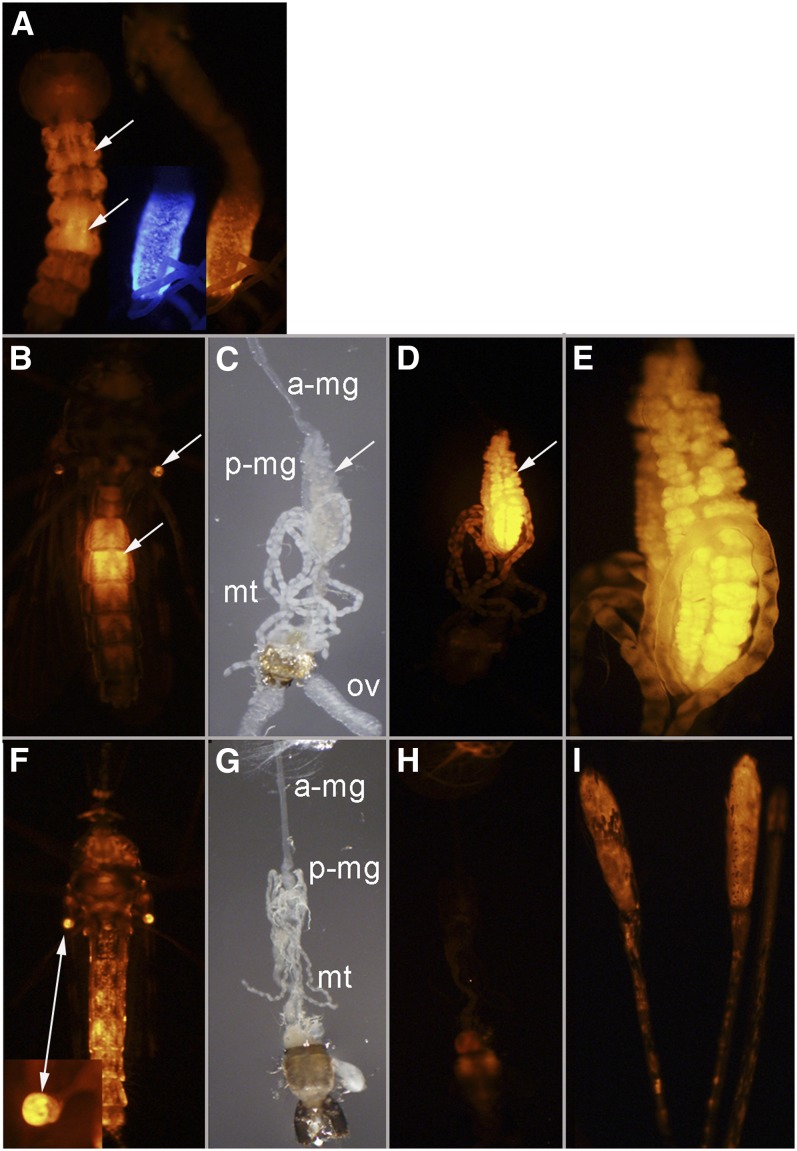
Line UMITF-C2F8. (A) Whole fourth-instar larva and dissected midgut. Arrows point to fat body and an intense region of *tdTomato* expression in the midgut. Dissected midgut shows overlapping patterns of expression of the *3xP3ECFP* marker gene associated with the *Gal4*-containing *piggyBac* element and the *UAStdTomato* transgene. The enhancer influencing *Gal4* expression is also having a similar effect on *3xP3ECFP*. (B) Ventral view of an unfed adult female. Arrows point to *tdTomato* expression in the abdomen and the distal region of the halteres. (C) Dissected alimentary canal of an unfed female. a-mg, anterior midgut; p-mg, posterior midgut (arrow); mt, Malpighian tubules; ov, ovaries. (D) Same alimentary canal as in (C) showing *tdTomato* expression only in the posterior midgut. (E) Close-up of the posterior midgut shown in (D). (F) Ventral view of an adult male with strong expression in the halteres (arrow) and abdomen. (G, H) Dissected alimentary canal of a male showing no *tdTomato* expression. (I) Maxillary palps from a male with strong *tdTomato* expression present at the terminal region.

*Gal4* is expressed strongly in the abdomen of larvae, including the fat body and a distinct region of the posterior midgut (Figure 3A). In this line, the enhancer is not only regulating expression of *Gal4* but also the *ECFP* marker gene that is under the regulatory control of the nerve-specific *3xP3* promoter. Although the *3xP3* promoter is known to be sensitive to enhancers, *tdTomato* expression and *ECFP* expression did not always overlap (see line UMITF-C2F41 below) (O’Brochta *et al.* 2011; Trauner *et al.* 2009). *Gal4* in this line was expressed in adult males and females (in Figure 3, compare B–E with F–I). In both sexes, strong *Gal4* expression was seen in the halteres (Figure 3, B and F). In females, aside from the halteres, *Gal4* expression was only detected in the posterior midgut both before and after blood feeding (Figure 3, C–E). No *Gal4* expression was detected in any other region of the alimentary canal, ovaries, or carcass. In adult males, the alimentary canal did not show any *Gal4* expression (Figure 3, G and H), although there was some expression associated with the abdominal epidermis (Figure 3F). Males also showed strong expression in the maxillary palps (Figure 3I).

#### UMITF-2MCL14:

**Figure 4:**
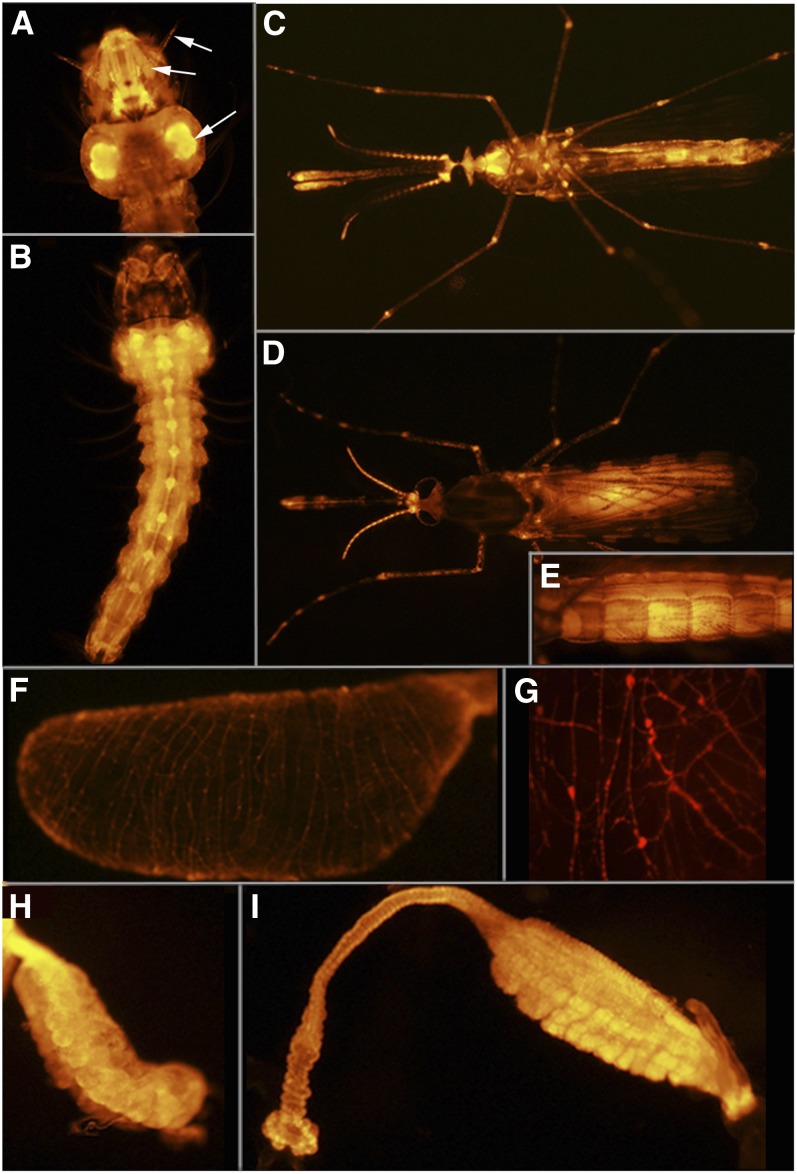
Line UMITF-2MCL14. (A) Dorsal view of the head and thorax of a fourth-instar larva with *tdTomato* expression in the musculature of the head, antennae, and salivary glands (arrows). (B) Ventral view of the same larva in (A) showing *tdTomato* expression in the musculature of the thorax and abdomen and the ventral ganglia. (C) Ventral view of an adult male with widespread expression throughout the body and notable expression in the maxillary palps, antennae, legs, thorax, and abdomen. (D) Dorsal view of an adult female with *tdTomato* expression resembling that seen in adult males. (E) Ventral view of the abdomen of the adult female in (D). (F) Crop of an adult female and (G) a close-up of same. (H) Previtellogenic ovaries. (I) Midgut of an adult female before blood feeding with *tdTomato* expression in the cardia, anterior midgut, and posterior midgut.

This line has widespread *Gal4* expression in both the larval and adult stages (Figure 4). In late instar larvae, *Gal4* expression occurs in some major muscle groups in the head, thorax, and abdomen, including muscles involved in moving mouthparts and longitudinal muscles extending down the ventral surface of the larva (Figure 4, A and B). *Gal4* expression is also seen in the ventral ganglia of the larva, in or around the salivary glands, and in the larval antenna (Figure 4, A and B). In adult males and females, widespread *Gal4* expression is seen in what appears to be neuronal tissue in the antennae, maxillary palps, and legs (Figure 4, C and D). The anterior and posterior midguts of unfed females express *Gal4* (Figure 4, E and I), as do cells of the crop (Figure 4, F and G) and previtellogenic ovaries (Figure 4H).

#### UMITF-C2F41:

**Figure 5:**
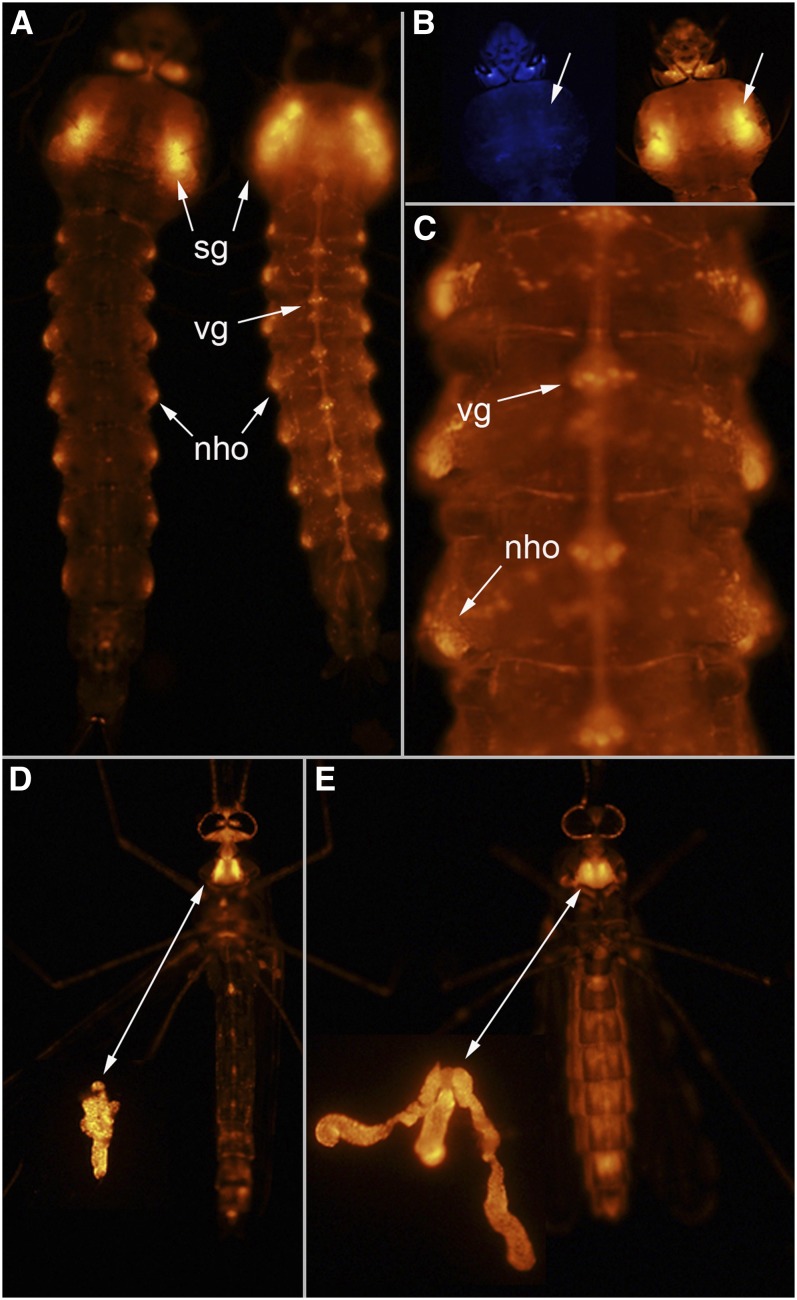
Line UMITF-C2F41. (A) Dorsal and ventral view of a fourth instar larva showing *tdTomato* expression in the salivary glands (sg), ventral ganglia (vg), and lateral neurohaemal organs (nho). (B) Dorsal view of the head and thorax of the fourth instar larva in (A) showing that the enhancer responsible for *Gal4* expression in the salivary glands does not influence the pattern of expression of *3xP3ECFP* (arrows). (C) Close-up of larval abdominal segments showing the ventral ganglia (vg) and lateral neurohaemal organs (nho). Ventral view of an adult male (D) and female (E) showing *tdTomato* expression in the head and the salivary glands. Arrows in (D) and (E) point to the salivary glands in the prothorax and following dissection.

Late instar larvae have *Gal4* expression in the salivary glands and some neuronal tissue, including the ventral ganglia, brain, and lateral structures that appear to correspond to neurohemal organs (Figure 5, A–C). In this line, expression of the *3xP3ECFP* marker gene is not influenced by the enhancer responsible for determining the observed pattern of *Gal4* as indicated by expression of *tdTomato* but not *ECFP* in the salivary glands (Figure 5B). In adults, both males and females have *Gal4* expression in the salivary glands and brain (Figure 5, D and E). The salivary glands of females have *Gal4* expression in the lateral and medial lobes (Figure 5E).

#### UMITF-2MCL6:

**Figure 6:**
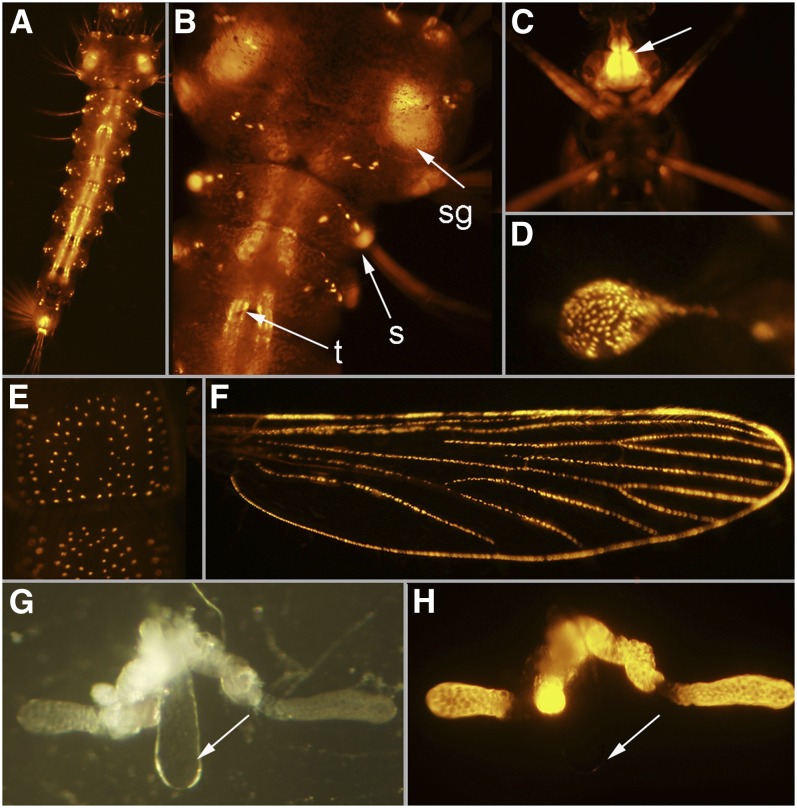
Line UMITF-2MCL6. (A) Dorsal view of a fourth instar larva. (B) Higher magnification of the fourth instar larva in (A) showing *tdTomato* expression in the salivary glands (sg), in cells at the base of the setae (s), and in the main trunk of the tracheal system (t). (C) Ventral view of an adult female showing *tdTomato* expression in the adult salivary glands (sg), visible through the cuticle of the episternum (arrow). (D) Haltere of an adult female. (E) Dorsal view of 1.5 abdominal segments of an adult female. (F) Wing of an adult female. (G) Salivary gland of an adult female showing the lateral lobes and the medial lobe (arrow). (H) Salivary gland in (G) showing *tdTomato* expression in only the proximal and distal lateral lobes.

*Gal4* is expressed in the larval salivary glands, cells at the base of larval setae, and cells in the main trunk of the tracheal system (Figure 6, A and B). Adults have *Gal4* expression in cells at the base of all scales and sensory bristles throughout the body (Figure 6, D–F). In addition, *Gal4* is expressed in the lateral lobes of the salivary glands of adult females, although there is no *Gal4* expression in the medial lobe (Figure 6, C, G, and H).

#### UMITF-AEA1:

**Figure 7:**
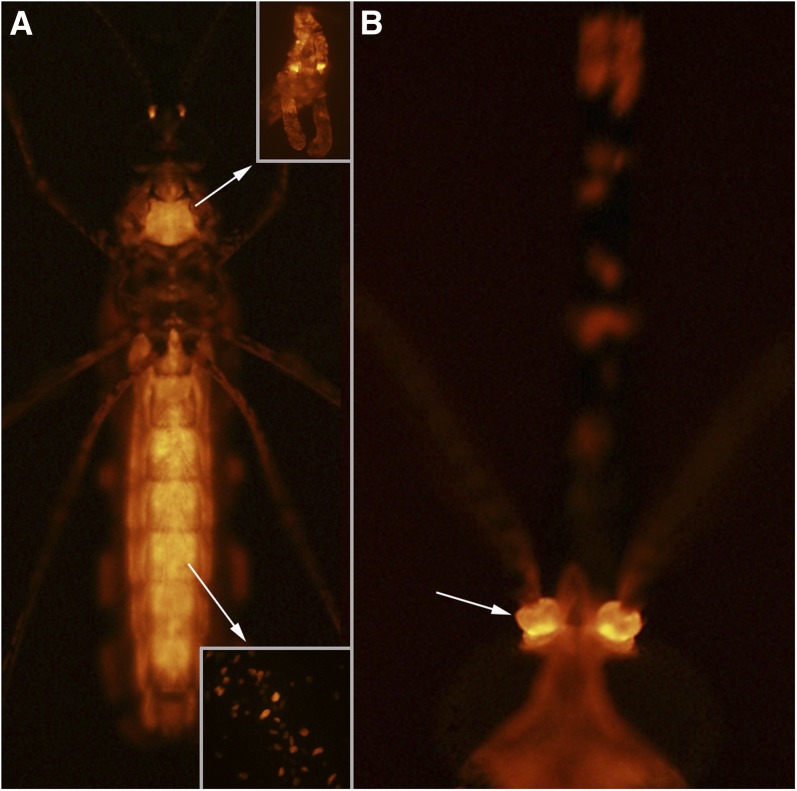
Line UMITF-AEA1. (A) Ventral view of an adult female with *Gal4* expression in the abdomen (arrow), thorax and salivary gland (arrow), and pedicel. (B) Dorsal view of the head of an adult female with *Gal4* expression in the pedicel of the antenna (arrow). Gal4 expression can also be seen in the maxillary palps, which are out of focus in this image.

No *Gal4* expression was detectable in the larval stages of this line. In adult females, *Gal4* expression was detected in abdominal fat body and weakly in the salivary glands (Figure 7A). *Gal4* was also expressed specifically in the pedicel at the base of the antenna of adults (Figure 7B).

#### UMITF-MBL24:

**Figure 8:**
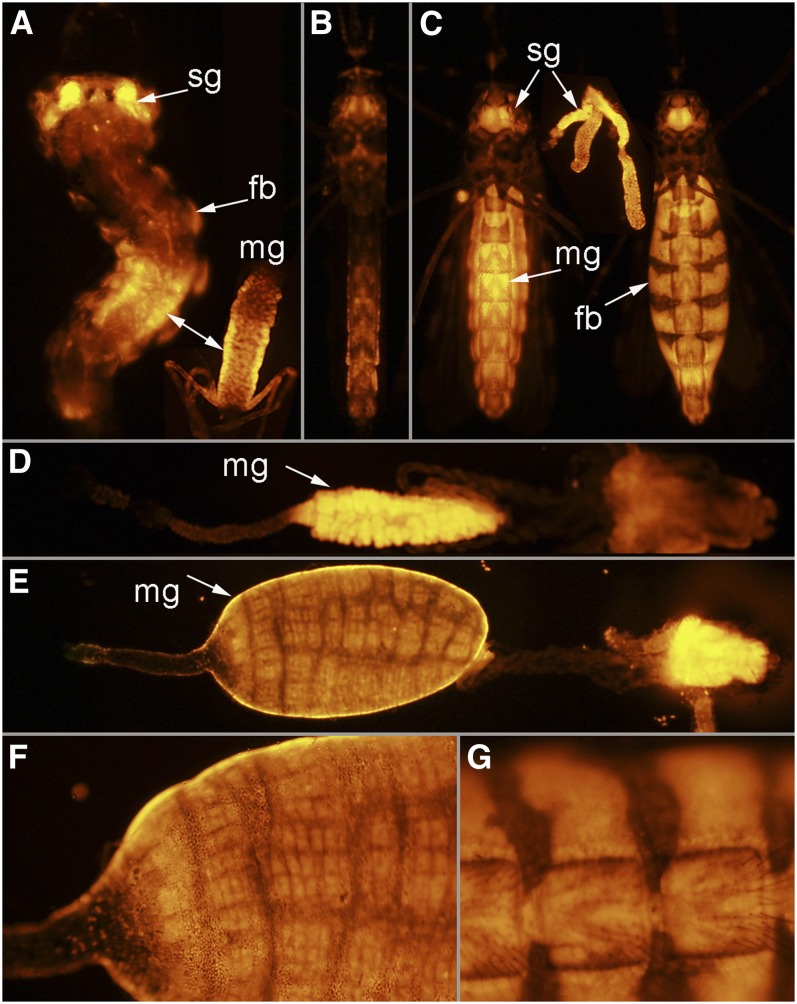
Line UMITF-MBL24. (A) A fourth instar larva with *tdTomato* in the salivary glands (sg), fat body (fb), and the posterior region of the midgut (mg). The double arrow points to the posterior region of the midgut of a fourth instar larva and of a dissected alimentary canal from a fourth instar larva. (B) Ventral view of an adult male. (C) Ventral view of an adult female before (left) and after (right) feeding. Dissected salivary glands are shown between images of adults. (D) Dissected alimentary canal of an unfed female with anterior to the left and posterior to the right showing the posterior midgut expressing *tdTomato*. (E) Dissected alimentary canal of a recently fed female with anterior to the left and posterior to the right showing the posterior midgut expressing *tdTomato*. (F) Close-up of midgut shown in (E). (G) Close-up of abdomen of the recently fed female shown in (C).

Larvae of this line have *Gal4* expression in the salivary glands, posterior midgut, and the abdominal fat body (Figure 8A). The patterns of *Gal4* expression in adult males and females strongly parallel the patterns of *Gal4* expression observed in the larval stages (Figure 8, B and C). The lateral and medial lobes of the adult female salivary glands strongly express *Gal4* (Figure 8C). In the midgut of both fed and unfed females, strong *Gal4* expression is observed specifically in the posterior midgut (Figure 8, C–F). The fat body in the female abdomen also strongly expresses *Gal4* (Figure 8, C and G).

#### UMITF-MDL8:

**Figure 9:**
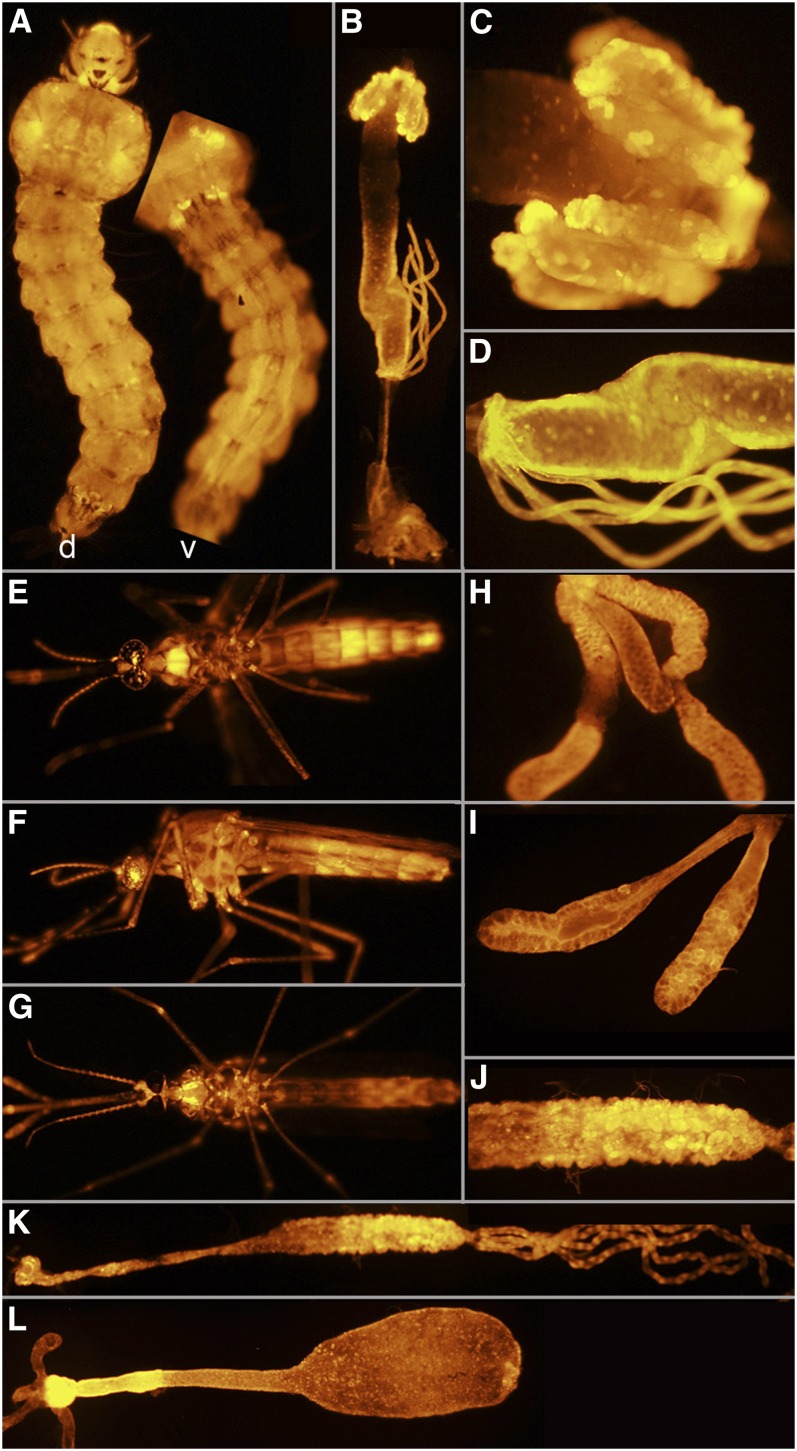
Line UMITF-MDL8. (A) Dorsal (d) and ventral (v) of a fourth instar larva showing widespread expression of *tdTomato*. (B) Dissected alimentary canal of a fourth instar larva showing *tdTomato* expression in the caecae, posterior region of the midgut, and the Malpighian tubules. (C) Higher magnification view of the caecae shown in (B). (D) Higher magnification of the posterior region of the midgut and Malpighian tubules. (E) Ventral view of an adult female. (F) Lateral view of the female in (E). (G) Ventral view of an adult male. (H) Salivary gland from an adult female. (I) Pre-vitellogenic ovaries. (J) Posterior region of the midgut from an adult female. (K) Alimentary canal of an adult female, including the cardia, anterior and posterior midgut, and Malpighian tubules. (L) Midgut of a female, post feeding.

*Gal4* is expressed throughout larvae, including muscles, fat body, and nervous tissue (Figure 9A). *Gal4* expression in the larval midgut is concentrated anteriorly in the caecae and in the posterior region of the midgut (Figure 9, B–D). Strong expression is also seen in the Malpighian tubules (Figure 9, B and D). Likewise in adult males and females, *Gal4* expression is widespread (Figure 9, E–G). Adult males and females have *Gal4* expression throughout the nervous system, including antennae, maxillary palps, legs, and brain (Figure 9, E–G). In adult females, all lobes of the salivary glands are expressing *Gal4*, as are the ovaries (Figure 9, H and I). *Gal4* expression is also seen in the alimentary canal, beginning with the cardia and including the anterior and posterior midgut and the Malpighian tubules (Figure 9, J and K). *Gal4* expression in the adult female midgut is not uniform, with distinctly more expression in the anterior midgut and cardia, as well as the posterior half of the posterior midgut (Figure 9K). *Gal4* expression in the adult female midgut is feeding independent (Figure 9L).

#### UMITF-2C2M8:

**Figure 10:**
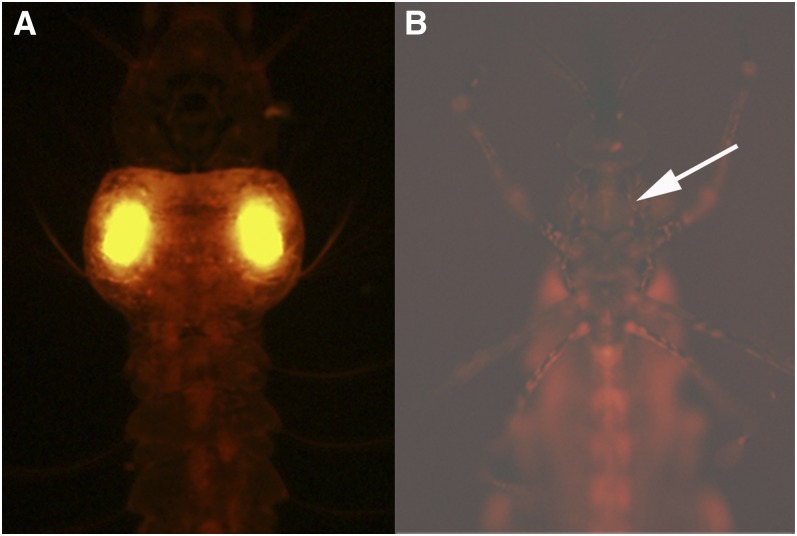
Line UMITF-2C2M8. (A) Dorsal view of a fourth instar larva with *tdTomato* expression only in the salivary glands. (B) Vental view of an adult female showing the absence of *tdTomato* expression in the salivary glands (arrow) and all other tissue.

*Gal4* expression in this line is restricted to only the salivary glands of larvae (Figure 10A). *Gal4* expression could not be detected in any other tissue in adult male or female mosquitoes (Figure 10B).

#### UMITF-DEA9A:

**Figure 11:**
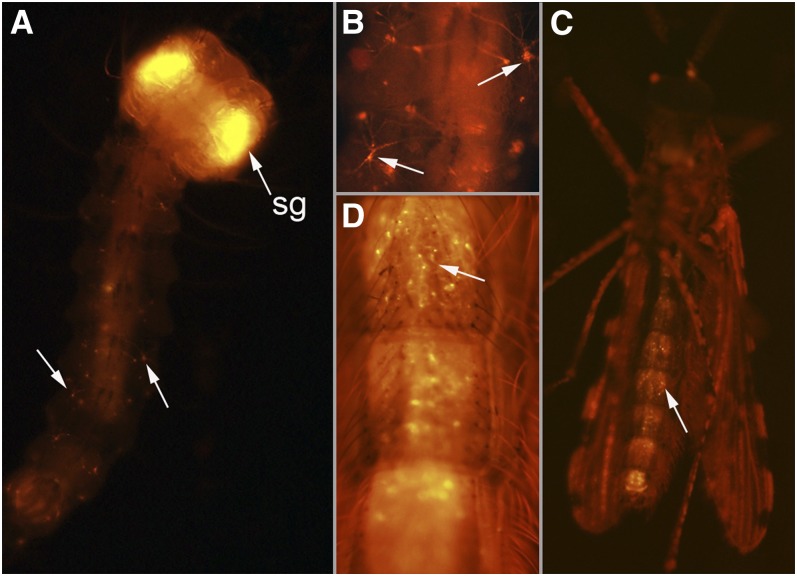
Line UMITF-DEA9A. (A) Dorsal view of a fourth instar larva with *tdTomato* expression in the salivary glands (sg) and in scattered stellate cells in the epidermis of the abdomen (arrows). (B) Higher magnification of the larval abdomen showing stellate cells expressing *tdTomato*. (C) Ventral view of an adult female with *tdTomato* expression in the abdomen. (D) Higher magnification of the abdomen of the adult female shown in (C) with scattered *tdTomato* expressing cells in or just under the epidermis.

*Gal4* expression is strongly localized to the salivary glands of larvae and in no other cells of the larva except for scattered stellate cells in the abdominal epidermis (Figure 11, A and B). In adults, *Gal4* expression is not observed in the salivary glands (Figure 11C), but there are isolated and evenly distributed cells in or just below the epidermis of the abdomen that express *Gal4* (Figure 11D).

#### UMITF-2MBL3:

**Figure 12:**
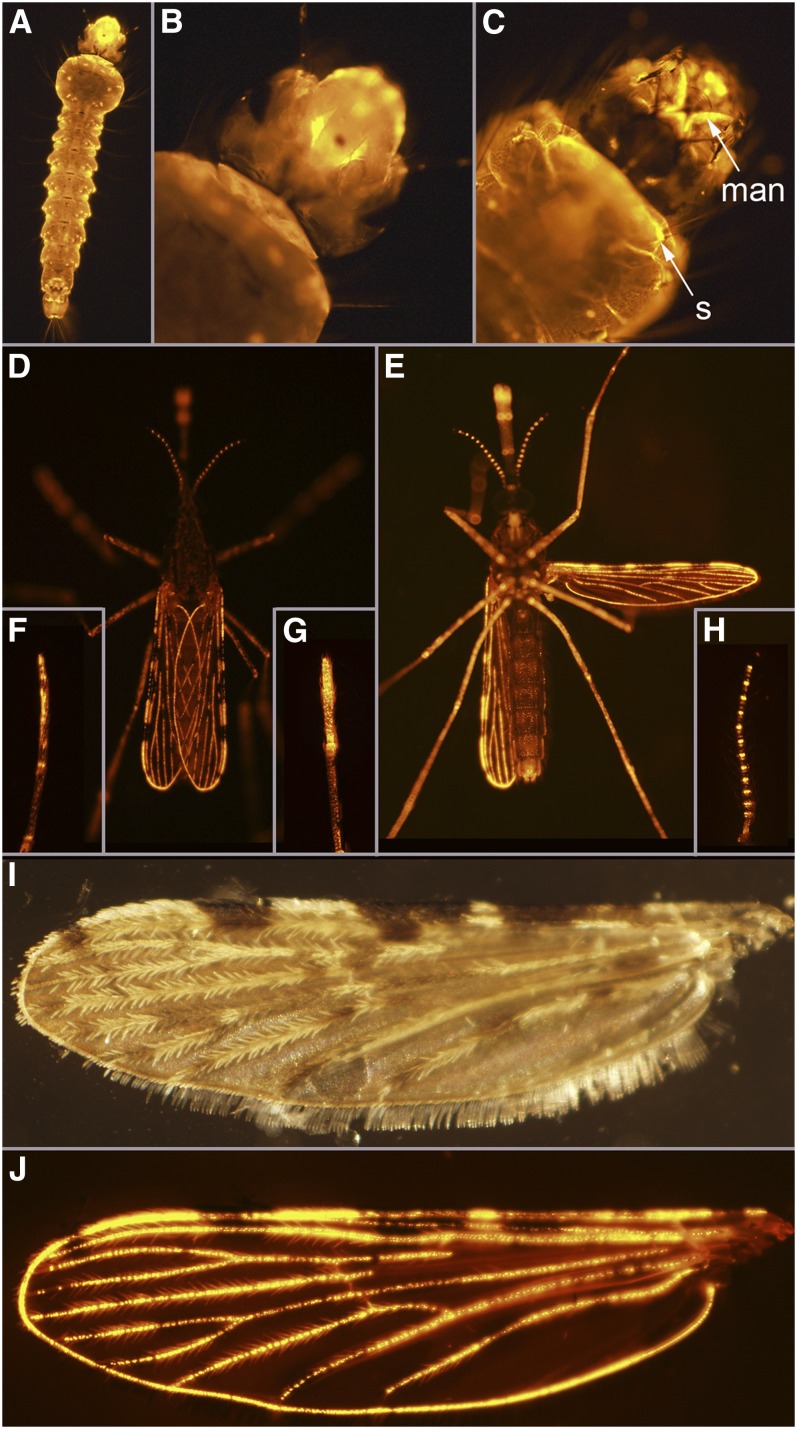
Line UMITF-2MBL3. (A) Dorsal view of a fourth instar larva. (B) Higher magnification of the dorsal side of the head of the larva in (A). No *tdTomato* expression is observed in the brain. (C) Higher magnification of the ventral side of the head of the larva in (B). *tdTomato* expression is seen in the setae associated with the mandibles (man) and at the base of setae (s) in the thorax. (D) Dorsal view of an adult female. (E) Ventral view of an adult female. (F) Tarsus from an adult female. (G) Maxillary palp from an adult female. (H) Antenna from an adult female. (I) Dorsal view of a wing from an adult female. (J) Same as (I) but under UV light to show *tdTomato*-expressing cells.

In both larvae and adults, *Gal4* expression in this line appears confined to a subset of cells in the peripheral nervous system (Figure 12). In larvae, this includes cells at the base of thoracic and abdominal setae, the larval antenna, and the setea lining the mandibles (Figure 12, A–C). *Gal4* expression is distinctly absent from the central nervous system, including the brain and ventral ganglia (Figure 12, B and C). A similar distribution of *Gal4* expression is seen in adults, with cells at the base of most setae, hairs, and scales strongly expressing *Gal4* (Figure 12, D–J). *Gal4* expression is seen in nerve-rich regions of the leg, maxillary palps, and antennae (Figure 12, F–H). Cells of the base of every scale on the wings express *Gal4* (Figure 12, I and J).

## Discussion

The functionality of the *Gal4/UAS* transcription regulatory system has been demonstrated in a range of eukaryotes, and when coupled to transposons, it becomes a powerful technology for the purposes of scanning genomes for the presence of gene regulatory elements and then using those regulatory elements to control transgene expression. Although useful, *Gal4*-based enhancer-trapping systems have been developed for few insects other than *D. melanogaster*, yet such systems powerfully complement existing efforts to manipulate insect genomes and determine the function of insect genes (Brand and Perrimon 1993; Trauner *et al.* 2009; Uchino *et al.* 2008). That such a system is now available for a major vector of human pathogens is of some significance given the interest in manipulating the genome of Anopheles mosquitoes not only for the purposes of advancing the functional genomics analysis of these insects but also for the development of novel strategies for controlling vector populations and their capacity to transmit parasites such as Plasmodium (Catteruccia 2007). The results presented here show that when coupled to *piggyBac* transposons and introduced into the genome of *An. stephensi*, the *Gal4/UAS* system can be used to readily detected enhancers with a wide variety of activities. In this system, the *piggyBac* transposase promoter located in the 5′ subterminal region of the element was used to provide essential basal promoter functions for *Gal4* gene regulation. This configuration of the *Gal4* enhancer-detection system in *An. stephensi* is similar to the *P*-element–based enhancer-trap system widely used in *D. melanogaster*, which utilizes the *P*-element transposase promoter to provide essential basal promoter functions for *Gal4* gene expression (Brand and Perrimon 1993). In both systems, the transposase promoters are weakly active and do not result in detectable levels of *Gal4* expression in larval or adult tissues in the absence of enhancers.

The frequency of enhancer detection in *An. stephensi* using the system described here was high enough to allow for the rapid generation and detection of enhancer-trap events. Of the approximately 24,000 progeny screened in this study as both larvae and adults that could potentially harbor an enhancer-trap event, approximately 300 were found with novel expression patterns of the reporter gene *tdTomato* due to the influence of an enhancer (317/24,250; 1.3%). As we reported in an earlier study (O’Brochta *et al.* 2011), we observed more *piggyBac* remobilization events when the system was in the germ line of females compared with males (Table 2). Although we observed these differences consistently and the differences were statistically significant, the biological basis and significance of these observations remain unknown, and additional data are needed to address this question. Because all matings in this study was performed *en masse*, we were unable to estimate the frequency of germ lines yielding enhancer-trap events; therefore, direct comparisons of the performance of this enhancer-trap system with the systems described for *Drosophila*, *Tribolium*, and *Bombyx* cannot be made (Trauner *et al.* 2009; Uchino *et al.* 2008). *Anopheles stephensi* is highly fecund in the laboratory, with females producing some 350 progeny over three gonotrophic cycles following blood meals, which means that genetic screens involving tens of thousands of progeny are practical. The number of progeny arising from each enhancer-trap event within a genome (sometimes referred to as “cluster size”) was very small in the genetic screens reported here. Multiple progeny with an identical pattern of *tdTomato* expression, containing *piggyBac* in the same genomic position and found among the progeny of a single genetic cross, were rarely recovered. At this point, the temporal patterns of *piggyBac* transposition within the germ line of *An. stephensi* are unknown, although our observations suggest that transpositions are not occurring early during germ line development. The promoter from the *hsp70* gene from *D. melanogaster* regulates the *piggyBac* transposase transgenes in lines F2 and M5; however, its expression did not require heat induction in *An. stephensi*. Future studies will explore the relationship between the timing and frequency of heat shock and the amount and timing of *piggyBac* remobilization and enhancer detection. Despite the fact that there are aspects of this enhancer-trap system that remain to be determined, it promises to be quite useful for creating *Gal4*-expressing *An. stephensi* lines with widespread utility.

The genetic manipulation of *An. stephensi* by vector biologists remains somewhat challenging because there are relatively few promoters that have been isolated and characterized, and the creation of transgenic *An. stephensi* remains technically demanding. Most transgenic lines of *An. stephensi* created to date have been single-purpose lines with limited utility beyond their intended function, which further increased the costs and inefficiencies associated with using transgenic technologies in this species. The development and use of a *Gal4*-based enhancer-trap system increases the utility of transgenic technologies in *An. stephensi* by providing researchers with many more options for expressing transgenes of interest in temporal and spatial patterns. For example, we described lines in which enhancers were detected that regulated *Gal4* expression in the adult female midgut, salivary gland, and fat body, three tissues that play critical roles in Plasmodium infection and transmission. Lines UMITF-C2F8 and UMITF-2MCL14 had *Gal4* expression in the midgut but not in the salivary glands or fat body of adult females. Lines UMITF-C2F41 and UMITF-2MCL6 had *Gal4* expression in the salivary glands but not in the midgut or fat body of adult females. Line UMITF-AEA1 had *Gal4* expression in the fat body but no expression in the midgut and only weak expression in the salivary glands of adult females. Line UMITF-MBL24 was particularly interesting from the perspective of Plasmodium infection of *An. stephensi* because *Gal4* expression occurred specifically in the adult female salivary glands, posterior midgut, and fat body. This line will permit transgenes to be expressed in three of the most important tissue compartments of *An. stephensi* with respect to Plasmodium infection within a single adult female. Line UMITF-MDL8 is expected to be useful because it has *Gal4* expression ubiquitously throughout most tissues of both larvae and adults. Although most of the lines reported here were chosen to illustrate the utility of this technology to the study of mosquito-parasite/pathogen interactions, lines UMITF-C2M8 and UMITF-DEA9A had *Gal4* expression exclusively or almost exclusively in larval tissue, whereas line UMITF-2MBL3 had *Gal4* expression in a specific subset of cells associated with scales and sensillae. The binary nature of this system permits the effort spent on creating transgenic lines to be minimized while enabling investigators repeated opportunities to express their transgene in a variety of patterns simply by mating their *UAS*-regulated transgene–containing line to any *Gal4*-expressing line. This modularity is perhaps the most important feature of this system.

The system described here, although effective at detecting enhancers, could be made more effective with two modifications. First, *piggyBac* transposase is currently not limited to the germ lines of lines M5 and F2. Because the transposition activity of the enhancer-trap element is sufficiently high in somatic cells, clones of *Gal4*-expressing cells in various tissues are frequently seen (Figure 2). If enhancer-trap events, in which the expected *Gal4* expression patterns involved relatively small numbers of cells resulting in a subtle but significant pattern of reporter gene expression, are of interest, then the somatic clones frequently observed with our current system could be a liability by making such patterns difficult to recognize. Limiting transposase expression to the germ line of *An. stephensi* could be accomplished by using regulatory sequences that result in germ line–specific transcription (Papathanos *et al.* 2009). Our current enhancer-trap system has also shown that having the *UAStdTomato* transgene in a *piggyBac* vector can be disadvantageous. For example, when *piggyBac* transposase is present, the *UAS*-containing *piggyBac* element can become unstable and be remobilized to new genomic locations. Also, performing splinkerette-PCR or using any other method to identify integration sites of *piggyBac* elements containing the enhancer-reporter can be confounded by the presence of *piggyBac* elements containing *UAS*-regulated reporter genes. Although this current system could be improved by incorporating all *UAS*-regulated reporter genes into vectors other than *piggyBac*, careful genetics and accounting for chromosomes containing the *piggyBac* transposase transgene will avoid any undesirable remobilization of other system components. Although having other transposon platforms upon which to build system components is convenient, the highly effective enhancer-trap systems created for *D. melanogaster* were based on a single transposon platform (Bellen 1999).

The abundance of genome information and the ease with which it can now be obtained makes the need eminent for technologies that enable progress to be made in pursuing questions relating to functional genomics. For Anopheles mosquitoes, there are relatively few tools available for empirically assessing gene function within the context of the whole organism. The enhancer-trap system described here is a valuable first step in increasing our capacity to explore the biology of Anopheles mosquitoes using forward and reverse genetic approaches.
